# External Validation and Evaluation of Reliability and Validity of the S-ReSC Scoring System to Predict Stone-Free Status after Percutaneous Nephrolithotomy

**DOI:** 10.1371/journal.pone.0083628

**Published:** 2014-01-08

**Authors:** Min Soo Choo, Chang Wook Jeong, Jae Hyun Jung, Seung Bae Lee, Hyeon Jeong, Hwancheol Son, Hyeon Hoe Kim, Seung-june Oh, Sung Yong Cho

**Affiliations:** 1 Department of Urology, Seoul National University Hospital, Seoul, Korea; 2 Department of Urology, Seoul National University Boramae Medical Center, Seoul, Korea; Eberhard-Karls University, Germany

## Abstract

**Objectives:**

The Seoul National University Renal Stone Complexity (S-ReSC) scoring system was developed to predict the stone-free rate (SFR) after single-tract percutaneous nephrolithotomy (PCNL). This study is an external validation of this scoring system.

**Materials and methods:**

A retrospective review included 327 patients who underwent PCNL at 2 tertiary referral centers. The S-ReSC score was assigned from 1 to 9 based on the number of sites involved. The stone free status was defined as either complete clearance or clinically insignificant residual fragments <4 mm in size at 1 month follow-up imaging. Inter-observer and test-retest reliabilities were evaluated. The statistical performance of the prediction model was assessed by its predictive accuracy, predictive probability, and clinical usefulness.

**Results:**

The overall SFR was 65.4%. SFRs were 83.9%, 47.6%, and 21.4% in low (1–2), intermediate (3–4), and high (5–9) score groups, respectively, with significant differences (*P*<0.001). Inter-observer and test-retest reliabilities revealed almost perfect agreements. External validation of the S-ReSC scoring system revealed an AUC of 0.731 (95% CI 0.675–0.788). The AUC of 3-titered S-ReSC score groups was 0.691 (95% CI, 0.629–0.753). The calibration plot showed that the predicted probability of SFR had a concordance comparable to that of the observed frequency. The Hosmer–Lemeshow goodness-of-fit statistic revealed an adequate performance of the predictive model (*P* = 0.10). Inter-observer and test-retest reliability showed a good level of agreement.

**Conclusions:**

The S-ReSC scoring system is useful in predicting the post-PCNL SFR and in describing the complexity of renal stones.

## Introduction

The incidence and prevalence of kidney stones is increasing globally, regardless of sex, race, and age [1]. With technical advances in endoscopic instrumentation, the number of percutaneous nephrolithotomy (PCNL) procedures has increased dramatically in the last decade [2], [3]. Overall stone-free rates (SFRs) after PCNL are estimated to range from 56% to 76% [4], [5]. Additionally, combination therapy with SWL or use of multiple access tracts has increased SFR, but these approaches also increase the potential risk of complications like bleeding or a prolonged hospital stay, with increased cost of care [6]–[10]. Thus, an accurate prediction of SFR after PCNL is important when considering surgical modalities and necessity of ancillary procedures.

Studies have reported predictive factors of SFR after PCNL, such as complete staghorn stones, presence of secondary calyceal stones, stones of high calcium phosphate composition, and low BMI [4], [11], [12]. Recently, few research groups have developed prediction methods, and demonstrated advantages of predicting surgical outcomes of PCNL [5], [13]. However, these methods were too complex to use or required proprietary software. Furthermore, these prediction models had not been externally validated with separate populations in consideration of individual variation for interpretation of the grades. Most recently, Jeong *et al*. published the Seoul National University Renal Stone Complexity (S-ReSC) scoring system for defining the complexity of a renal stone and surgery [14]. The S-ReSC scoring system is simple to use and is precise at predicting the SFR after PCNL. The internal validation showed that the S-ReSC predicted SFR after PCNL accurately.

This scoring system must be validated externally before clinically to be used. Therefore, the present study is an attempt to validate the S-ReSC scoring system with an independent external cohort. Furthermore, the present study evaluated inter-observer and test-retest reliablity of the S-ReSC scoring system.

## Materials and Methods

### Subjects

A retrospective review of medical records and radiological imaging was performed for patients who had undergone single-tract PCNL for renal calculi at 2 tertiary referral centers between January 2004 and July 2012. Patients without preoperative computed tomography (CT) scan were excluded. Cases in which patients underwent PCNL with flexible nephro- or ureteroscope were also excluded. A total of 327 cases were included in the analysis. The study design and the use of patients' information stored in the hospital database were approved by the institutional review board at the Seoul National University Hospital (H-1210-123-437) and by the institutional review board at Seoul National University Boramae Medical Center (16-2013-11), and were performed in strict accordance with the ethical guidelines of the Declaration of Helsinki. We were given exemption from getting informed consents by the IRB because the present study is a retrospective study and personal identifiers were completely removed and the data were analyzed anonymously.

### Surgical methods

The details of PCNL procedure were described in previous publications [14]. With patients in prone position, a percutaneous nephrostomy tube was inserted by an experienced urologist or an uroradiologist. A calyceal puncture was usually performed at the lower-posterior calyx with a 22-gauge Skinny Needle (Cook Medical, Bloomington, IN, USA). The puncture tract was dilated with a 30-Fr balloon dilatation catheter (Nephromax™, Boston Scientific Corporation). The renal stones were fragmented by a lithoclast or Holmium:YAG laser (Trimedyne Inc., Irvine, USA). A 24-Fr nephroscope (Karl Storz, Tuttlingen, Germany) was inserted into the sheath, and the fragmented renal stones were retrieved. A 6-Fr urethral JJ stent was usually inserted and a 16-Fr urethral Foley catheter was placed into the urethra at the conclusion of procedure. Patients were discharged 2 or 3 days after procedures. The percutaneous nephrostomy tube and urethral Foley catheter were removed before discharge. The ureteral JJ stent was removed at the 2 week follow-up visit.

### Clinical parameters

Each patient's medical record was reviewed for medical history, physical examination, urinalysis, complete blood count, serum biochemistry, and coagulation tests. Preoperative non-enhanced CT images were evaluated for stone characteristics. The largest stone diameter was measured, and the stone volume was calculated by the ellipsoid formula (π/6×D3). The total volume was defined as the sum of individual stone volumes. The patients had been routinely assessed for any residual stones by follow-up plain kidney-ureter-bladder radiography or by CT scan in selected cases (i.e. radiolucent stones). Stone-free status was defined as either complete clearance or clearance with clinically insignificant residual fragments (<4 mm) at 1 month follow-up imaging [15].

### S-ReSC Scoring system

The S-ReSC scoring system counts the number of sites involved and they did not consider the size or number of stones. The potential sites of involvement were categorized into renal pelvis (#1), superior and inferior major calyceal groups (major calyx and infundibulum) (#2-3), and anterior and posterior minor calyceal groups of the superior (#4-5), middle (#6-7), and inferior calyx (#8-9) [14]. At each site, the presence of a stone, or multiple stones, was worth a single point such that the S-ReSC score ranged from 0 to 9, where 0 signified no stones in the entire kidney, and 9 signified stones in every one of the 9 sites. Because all of the patients had at least one kidney stone, the actually S-ReSC scores ranged from 1 to 9 in this study. The scores were categorized into 3 subgroups: low (1–2 points), intermediate (3–4 points), and high (5–9 points) score groups.

### External validation, inter-observer and test-retest reliabilities

To evaluate inter-observer agreement, a junior faculty member, a fellow, a junior resident, and a surgical assistant nurse participated in appraising S-ReSC scores of each patient. First of all, one junior faculty member (Cho S.Y.) evaluated the images and rated the S-ReSC scores from 1 to 9 for all patients. Among the 327 patients, a total of 45 cases, per 5 cases for each of the points, were selected for inter-observer and test-retest reliabilities. The remaining participants underwent 30-minute self-training sessions in calculating S-ReSC scores. Test-retest reliability was assessed over a period of 2 weeks. The intraclass correlation coefficients and Cohen's kappa were analyzed to evaluate inter-observer and test-retest reliabilities.

Continuous variables were presented as mean ± SD. The statistical performance of the prediction model was assessed by predictive accuracy, predictive probability, and clinical usefulness. The area under the curve (AUC) of receiver-operating curve was used to assess the predictive accuracy for SFR. Calibration plot was created to demonstrate the relationship between predicted and observed SFR with using 200 bootstrap resamples. A decision curve analysis was constructed to test the clinical utility of the prediction model. Univariate and multivariate logistic regression analyses were performed to identify significant predictors of stone-free status. A two-sided *P* value less than 0.05 was considered statistically significant. All statistical analyses were conducted by using SPSS (18.0 Inc., Chicago, IL, USA) and The R Project (i386 3.0.1) for Windows, version 2.15.2 (http://www.r-project.org/).

## Results

### Patient characteristics

The table 1 lists the characteristics of the 327 patients enrolled in the validation data set and the 155 patients in the original development data set. The clinical parameters showed no significant differences between the two groups. The table 2 summarizes SFRs according to S-ReSC scores. The overall SFR was 65.4% for the 327 patients. The SFR gradually decreased from 86.0% (score 1) to 0% (score 9) with decreasing S-ReSC score. When the S-ReSC scores were treated as a continuous variable, the odds ratio for each one-point increase was 1.44 (95% CI, 1.23–1.67). When patients were stratified into three subgroups according, there were 191 (59.5%), 96 (29.9%), and 34 (10.6%) in low (1–2), intermediate (3–4), and high (5–9) score subgroups, respectively. SFRs were 83.9%, 47.6%, and 21.4% in low, intermediate, and high score group, respectively, with significant differences between the groups (*P*<0.001).

**Table 1:** Comparison of patients' demographics between the validation data and the original development data of S-ReSC scoring system.

		Validation group	Development group	*P* value
No. of patients		327	155	
Age, year		53.6±13.3	54.9±13.6	0.905
Body mass index, kg/m^2^		25.5±7.8	25.5±3.6	0.224
Gender, No. (%)				0.449
	Male	220 (68.5)	101 (65.2)	
	Female	101 (31.5)	54 (34.8)	
Previous treatment, No. (%)		42 (13.0)	52 (33.5)	0.398
	Shock wave lithotripsy	27 (8.4)	35 (22.6)	
	Retrograde intrarenal surgery	11 (3.4)	5 (3.2)	
	Percutaneous nephrolithotomy	4 (1.2)	12 (7.7)	
Laterality, No. (%)				0.673
	Right	163 (50.8)	76 (49.0)	
	Left	158 (49.2)	19 (51.0)	
Number of stone, No. (%)				0.256
	1	110 (34.3)	88 (56.8)	
	2	57 (17.8)	28 (18.1)	
	3	19 (5.9)	8 (5.2)	
	4+	135 (42.0)	31 (20.0)	
The largest diameter, mm		23.2±9.4	23.6±9.2	0.356
Total stone volume, cm^3^		5.87±91.2	13.5±21.9	0.113
Major stone composition, No. (%)				0.656
	Calcium oxalate monohydrate	78 (23.9)	75 (48.4)	
	Calcium oxalate dehydrate	7 (2.14)	21 (13.5)	
	Calcium phosphate	3 (0.9)	5 (3.2)	
	Carbonite apatite	16 (4.9)	7 (4.5)	
	Uric acid	23 (7.0)	23 (14.8)	
	Struvite	15 (4.6)	9 (5.8)	
	Cystine	2 (0.6)	2 (1.3)	
	others	11 (3.4)	11 (7.1)	
	missing	172 (52.6)	2 (1.3)	

**Table 2:** Stone-free rates according to S-ReSC scoring system.

S-ReSC score	Stone-free rate	S-ReSC group	Stone-free rate	*P* value	OR	95% CI
1	86.0% (92/107)	Low (1–2)	83.9% (151/180)	Reference		
2	80.8% (59/73)					
3	50.7% (36/71)	Intermediate	47.6% (50/105)	<0.001	0.17	0.10–0.30
4	41.2% (14/34)	(3–4)				
5	25.0% (4/16)	High (5–9)	21.4% (9/42)	<0.001	0.02	0.02–0.12
6	30.8% (4/13)					
7	0% (0/2)					
8	20% (1/5)					
9	0% (0/6)					
	*P*<0.001		*P*<0.001			

OR, Odds ratio; CI, confidence interval.

### Inter-observer and test-retest reliability

As shown in Table 3, inter-observer reliability for the S-ReSC scoring system demonstrated almost perfect levels of agreement among the graders. The intraclass correlation coefficients were 0.949 (95% CI 0.922–0.969, *P*<0.001). Test-retest reliability showed almost perfect levels of agreement for most graders. The surgical assistant nurse demonstrated a substantial agreement between S-ReSC scores. All values for Cohen's kappa ranged from 0.768 to 0.994.

**Table 3:** Inter-observer and test-retest reliability of S-ReSC scores over a period of 2 weeks.

	Inter-observer agreement		Test-retest reliability	
	Crohnbach's alpha	*P* value	Cohen's Kappa	*P* value
	(95% CI)		(95% CI)	
Junior faculty	0.949 (0.922–0.969)	<0.001	0.978 (0.938–0.994)	<0.001
Fellow			0.962 (0.932–0.979)	<0.001
Resident			0.994 (0.989–0.997)	<0.001
Assistant nurse			0.768 (0.580–0.872)	<0.001

CI, confidence interval.

### Prediction accuracy: discrimination, calibration, and clinical usefulness

**Figure 1:**
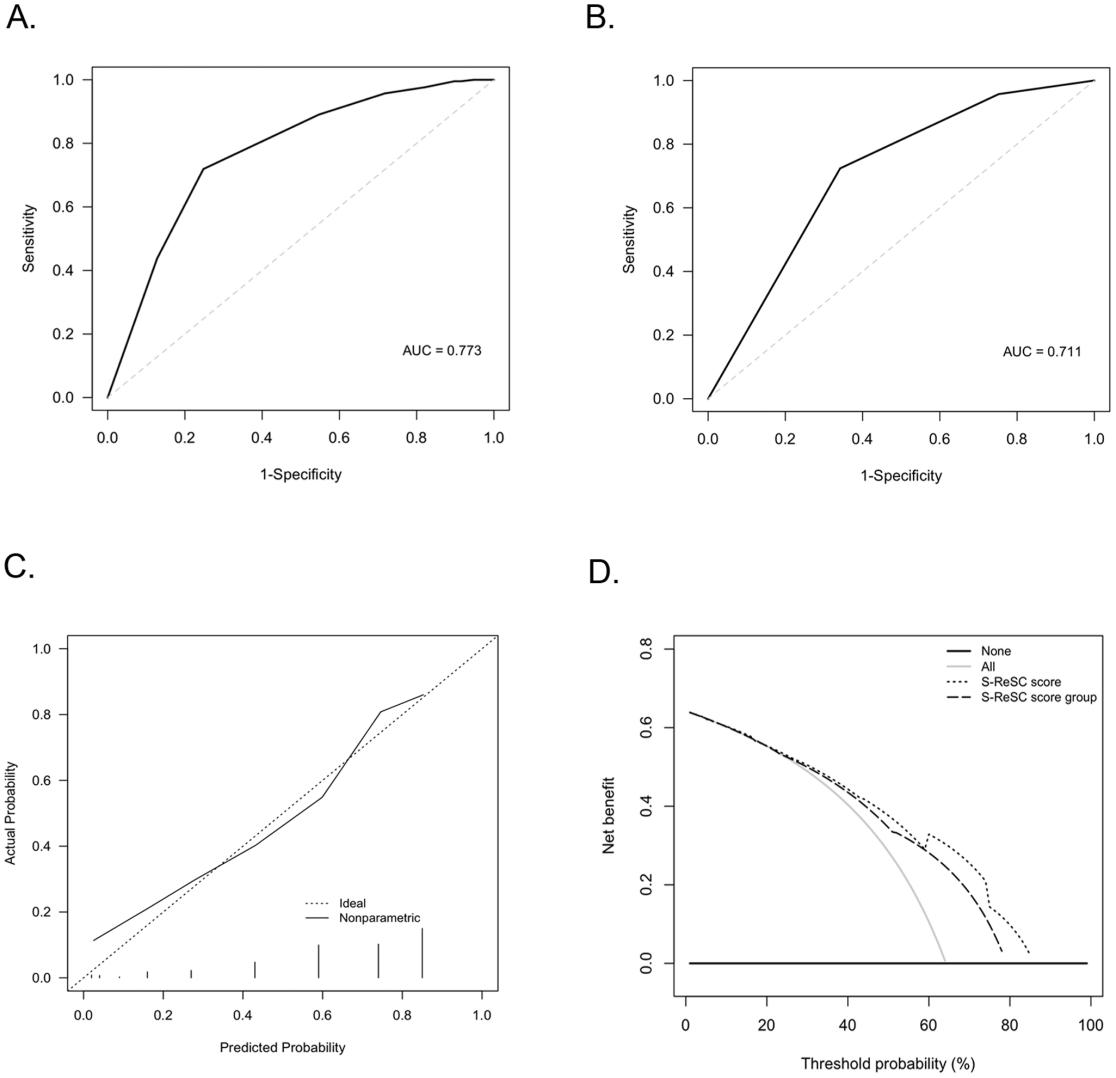
external validation of S-ReSC scoring system. A, ROC curve of S-ReSC scoring system. B, ROC curve of 3-titered S-ReSC score subgroups. C, a calibration plot to show the predicted probability of stone free rate. D, a decision curve analysis revealed that the prediction model provided a superior net benefit using S-ReSC scoring system.

As shown in Figure 1A, external validation of S-ReSC scoring system revealed an AUC of 0.773 (95% CI 0.719–0.828). The AUC of 3-titered S-ReSC score subgroups was 0.759 (95% CI, 0.703–0.815) (Figure 1B). As shown in Figure 1C, the calibration plot showed that the predicted probability of SFR had concordance comparable to that of the observed frequency, with most predictions within a 5% margin of error. The mean absolute error was 0.038 in S-ReSC scoring system and 0.020 in the S-ReSC score subgroups. The Hosmer-Lemeshow goodness of fit test revealed a *P* value of 0.64 for S-ReSC scoring system and 0.83 for the S-ReSC score subgroups, indicating a good logistic regression model fit. In decision curve analysis, the prediction model provided a superior net benefit and reduction with a probability threshold of around 20% (Figure 1D).

### Uni- and multivariate logistic regression analyses for predictors of stone-free status

In univariate logistic regression analysis, age, S-ReSC score, stone number, largest diameter, and total stone volume were significant predictors for SFR. Multivariate logistic regression analysis indicated that only S-ReSC score and number of stones were significant predictors for SFR (Table 4).

**Table 4:** Uni- and multivariate logistic regression analyses for predictors of stone-free status.

		Univariate			Multivariate		
		OR	95% CI	*P* value	OR	95% CI	*P* value
Age		0.981	0.964–0.999	0.036			
BMI		0.991	0.931–1.055	0.775			
Laterality		0.695	0.440–1.098	0.119			
S-ReSC score group							
	Low vs intermediate	0.197	0.114–0.338	<0.001	0.376	0.187–0.756	0.006
	Low vs high	0.133	0.064–0.276	<0.001	0.161	0.064–0.402	<0.001
Stone number							
	1 versus 2	0.183	0.080–0.417	<0.001	0.126	0.050–0.319	<0.001
	1 versus 3	0.400	0.113–1.421	0.157	0.260	0.068–1.001	0.050
	1 versus 4+	0.083	0.041–0.169	<0.001	0.127	0.055–0.296	<0.001
Largest diameter		0.961	0.937–0.985	0.001			
Total stone volume		0.999	0.998–0.999	<0.001			

OR, Odds ratio; CI, confidence interval; BMI, body mass index.

## Discussion

### Development of scoring systems for prediction of SFR

To reliably extract renal stones, understanding the anatomy of complex renal collecting system is important [16]. Previous investigations have developed several scoring systems for predicting surgical outcomes [13], [17]. ‘Guy’s stone score' included stone number, location, presence of staghorn stone, and abnormal anatomy in the determination of grades, and SFR decreased according to increasing grades [13]. Grades I and II differentiated stone locations between upper pole and other poles, which reflected surgical difficulties in previous investigations [5]. However, this scoring system does not appear to be widely used because it does not allow for an immediate determination of the grades. Staghorn morphometry is a new prediction model for PCNL [17], which was based on accurate measurement of stone volume. However, that model requires the proprietary software which is not openly available. Additionally, surgical difficulty in the extraction of renal stones has usually been associated with the complex anatomy of renal collecting system, which is not necessarily related to the volume of stone.

### S-ReSC scoring system, its pros and cons

The S-ReSC scoring system was developed to predict SFR after PCNL. The difficulty in complete removal of stones are related to many factors such as stone size and volume, stone distribution, the number of stone, and anatomy of renal collecting system. Fundamentally, the S-ReSC scoring system was developed with the hypothesis that the distributional complexity of stones was the most powerful predictor of SFR. However, this system is not based solely on these factors because stone distributions are closely related to stone size, volume, and stone number.

This scoring system may have some disadvantages. It cannot demonstrate whether there are differences in SFR among stones of different sizes in the same calyx or not, nor can it explain whether there are differences in SFR among stones of different sizes in the different calyces, one of which may have an abnormal anatomy. Despite these disadvantages, the S-ReSC scoring system is very easy to use, quantitative, accurate and reproducible. Additionally, all graders were able to reliably appraise the S-ReSC scores. The scores accurately predict SFR after PCNL and, in the original study describing S-ReSC, the AUC was reported to be 0.86 for predicting SFR.

In the previous study, the authors had performed internal validation of the S-ReSC scoring system. Validation using an external data set is a stringent test of a prediction model [18]. For this purpose, the data of four expert surgeons from two independent institutions were used. The result from this study revealed a reasonable discrimination ability with an AUC of 0.773 and confirmed that this prediction model can accurately estimate the probability of SFR after PCNL. Thus, this new prediction model may be helpful in the evaluation of patients with renal stone who are being considered for ancillary procedures to achieve stone-free status. This AUC with the validated data set was lower than that of the original developed data set (0.860 versus 0.773, respectively). This may be caused by differences in the respective populations.

### Removal of stones in upper calyces

The S-ReSC scoring system does not take into account upper pole calyceal stones, which are generally thought to be more difficult to remove than stones in other positions with PCNL [5]. Therefore, the supracostal access was frequently necessary for removal of the upper pole calyceal stones, and this approach increased the risk of intrathoracic complications [7], [10]. Munver et al. reported that the incidence of intrathoracic complications with a supra-11^th^ rib access was 23.1%, which was 16-fold greater than the rate of 1.4% for a supra-12^th^ rib access and 46-fold greater than the 0.5% for a subcostal access [10]. In our study, the SFR for upper pole calyceal stone was significantly lower than that for other locations (31.3% versus 74.9%, *P*<0.001). If the upper pole calyceal stones were considered as an additional one (1) point in the S-ReSC appraisal, the AUC increased from 0.773 to 0.788. However, this was not a significant increase. This may be because 87.5% of upper pole calyceal stones were over 3 points of S-ReSC score, which meant that upper pole calyceal stones would have inherently more complex characteristics.

### Limitations of the present study

This present study had several limitations. The retrospective nature of the design renders if vulnerable to a selection bias. However, the effect on the results was minimal because our data was consecutively collected in two independent institutions by 4 surgeons. Another limitation was that the CT scan protocols were not standardized. Most CT images had been obtained with 3.0-mm section thickness, but some were obtained with 2.5-mm or 5.0-mm thickness. Theoretically, these differences might have led to different SFRs; however, this must have been rare because the presence of stones was double-checked with plain X-ray films.

## Conclusion

The present study confirms the predictive value of S-ReSC scoring system to predict stone-free status after PCNL in an independent cohort. Inter-observer and test-retest reliabilities demonstrated the S-ReSC scoring system to be reliable and valid. Further investigations with larger study samples are needed to evaluate the clinical significance of this scoring system.
